# Melatonin Enhances Drought Tolerance by Regulating Leaf Stomatal Behavior, Carbon and Nitrogen Metabolism, and Related Gene Expression in Maize Plants

**DOI:** 10.3389/fpls.2021.779382

**Published:** 2021-12-13

**Authors:** Chengfeng Zhao, Haoxue Guo, Jiarui Wang, Yifan Wang, Renhe Zhang

**Affiliations:** College of Agronomy, Northwest A&F University, Yangling, China

**Keywords:** maize, drought, melatonin, stomatal behavior, carbon and nitrogen metabolism

## Abstract

It is commonly known that exogenously applied melatonin can alleviate the impact of drought stress, but the mechanism used by melatonin to regulate stomatal behavior and carbon (C) and nitrogen (N) metabolism to increase drought resistance remains elusive. Herein, our aim was to investigate the influence of exogenous melatonin on the regulation of C and N metabolism in maize plants under water deficit. In this study, we analyzed stomatal behavior, the key components of C and N metabolism, and the gene expression and activity of enzymes involved in the C and N metabolism in maize plants. The results showed that the application of melatonin (100 μM) significantly increased maize growth and sustained the opening of stomata, and secondarily increased the photosynthetic capacity in maize. Under drought stress, foliar application of melatonin induced the gene transcription and activities of sucrose phosphate synthetase, ADP-glucose pyrophosphorylase, phosphoenolpyruvate carboxylase, and citrate synthase, resulting in the enhancement of sucrose and starch synthesis and the tricarboxylic acid (TCA) cycle. This enhancement in sugar biosynthesis and the TCA cycle might lead to stronger N assimilation. As anticipated, NO_3_^–^ reduction and NH_4_^+^ assimilation were also strengthened after melatonin treatment under drought stress. An increase was observed in some key enzymatic activities and transcription involved in nitrogen metabolism, such as that of nitrate reductase, nitrite reductase, glutamate synthase, and glutamine synthetase, in melatonin-treated, drought-stressed maize. Moreover, melatonin attenuated the drought-induced damage by reducing protein degradation and increasing the level of proline. Conclusively, our results indicate that exogenous melatonin enhances drought tolerance in maize *via* promoting stomatal opening and regulating C and N metabolism and related gene expression.

## Introduction

Maize (*Zea mays* L.) is one of the most important grain crops cultivated worldwide but is extremely sensitive to drought stress (Li Z. et al., 2021). Water is a crucial environmental factor for crop production, and soil water deficits limit crop growth and yield (Yang et al., 2019). Water scarcity compromises economic output and food security worldwide, and in the past decade, global losses in crop production due to drought totaled approximately $30 billion (Riemann et al., 2015; Gupta et al., 2020).

Drought is complex abiotic stress, and a series of morphological, physiological, and biochemical changes take place during the response of plants to drought stress (Shah et al., 2020), which include plant growth (Todaka et al., 2017), leaf stomatal behavior (Indira et al., 2021), photosynthetic activity (Zhou et al., 2019), cellular redox homeostasis (Zhang et al., 2019), and metabolism homeostasis (Pinheiro and Chaves, 2011). These changes are usually interconnected. Specifically, osmotic stress caused by drought induces the accumulation of abscisic acid (ABA), which, at high levels, can promote stomata closure and decrease the internal carbon dioxide concentration (Ci) (Riemann et al., 2015; Kong et al., 2016; Zhou et al., 2019). Following stomatal closure and the decrease in Ci, the activity of the carboxylating enzyme Rubisco has been shown to decrease, which leads to electron accumulation and reactive oxygen species (ROS) overproduction, eventually resulting in oxidative damage and a series of subsequent side effects, such as leaf peroxidation, and degradation of chlorophyll, proteins, and nucleic acids (Campos et al., 2019; Sharma and Zheng, 2019; Sharma et al., 2020).

To cope with drought stress, the plants have evolved various metabolic adaptation mechanisms to defend against the adverse effects of stress, in which the coordinated regulation of carbon (C) and nitrogen (N) metabolism is one of the most important mechanisms (Ren et al., 2020). C and N metabolism are two of the most important metabolic processes in plants, and they are tightly related to each other (Yu et al., 2021). Metabolic processes involving C include reactions in photosynthesis and respiration (Cui et al., 2019). Photosynthesis and mitochondrial respiration provide C skeletons and an energy source for various biological processes, such as N assimilation and amino acid biosynthesis (Qiao et al., 2019). The growth and yield of plants are determined to a large extent by the capacity of photosynthesis (Cui et al., 2019). However, water deficit limits photosynthesis, which causes depletion of energy and sugar and diminishes plant production (Hu et al., 2020). N is a crucial structural component of nitrogenous compounds, such as amino acids, proteins, nucleic acids, chlorophyll, and enzymes (Qiao et al., 2019). Thus, N directly or indirectly affects plant photosynthesis through its effects on chlorophyll, photosynthetic rate, and the main enzymes of dark reactions and photorespiration (Zhong et al., 2019). The first step in N uptake and utilization is that nitrate reductase (NR) and nitrite reductase (NiR) convert nitrate (NO_3_^–^) into ammonium (NH_4_^+^) (Xie et al., 2019). Then, NH_4_^+^ is further assimilated to glutamate *via* glutamine synthetase (GS) and glutamate oxoglutarate aminotransferase (GOGAT) or glutamate dehydrogenase (GDH) (Rajasekhar and Oelmüller, 2010; Xie et al., 2019). Subsequently, glutamate acts as a donor of the amino group that distributes N to all other N-containing metabolites and macromolecules (Xie et al., 2019). The studies have shown that N assimilation plays a pivotal role in the acclimation of plant photosynthesis to drought stress (Xie et al., 2019; Zhong et al., 2019). Moreover, the C metabolism provides the energy and organic carbon skeletons for N assimilation and amino acid biosynthesis (Zhong et al., 2019). Therefore, the balance between C and N metabolism provides essential contributions to drought tolerance (Liu et al., 2014; Ren et al., 2020).

Melatonin (N-acetyl-5-methoxytryptamine) is a new plant growth regulator that is widely found in bacteria, fungi, plants, and algae (Kanwar et al., 2018; Debnath et al., 2020). Previous reports demonstrated that melatonin is involved in multiple biological processes in plants, such as seed germination (Li C. et al., 2021), root growth (Boyko et al., 2020), flowering (Kolar et al., 2003), leaf senescence (Ahmad et al., 2020), increased photosynthetic capacity (Ahmad et al., 2019), and moderation of oxidative damage (Qi et al., 2018; Kaya et al., 2019, 2020; Siddiqui et al., 2020). Furthermore, many studies have shown that the antioxidant action of melatonin can substantially enhance the tolerance of plants under biotic and abiotic stresses, such as pathogen infections (Li et al., 2019), drought (Sharma et al., 2020), cold (Wang et al., 2020), heat (Wei et al., 2015), salt, and UV stress (Yao et al., 2020; Zhang et al., 2020). In addition, melatonin may enhance plant stress resistance by regulating C or N metabolism. A previous study has suggested that metabolites, such as carbohydrates, organic acids, and amino acids accumulate after the application of melatonin to increase cold stress tolerance in Bermuda grass (Hu et al., 2016). A recent study in cotton revealed that melatonin enhances pollen fertility by balancing the carbohydrates of drought-stressed anthers (Hu et al., 2020). However, most of these studies on melatonin-enhancing stress resistance focused only on C or N metabolism, and currently, there is no report that combined C and N metabolism to study how melatonin alleviates drought stress.

Given the essential contributions of C and N metabolism and melatonin to the drought resistance of maize and the regulatory role of melatonin on primary metabolism, we hypothesize that the melatonin-induced drought resistance of maize depends to a large extent on the coordinated modulation of C and N metabolism. Therefore, we investigated the possible role of melatonin in maize response to soil drought stress by determining the photosynthetic capacity, leaf stomatal behavior, the amounts of various metabolites related to C and N metabolism, and the gene expression and activities of some key enzymes involved in C and N metabolism. The current study aimed to explore how melatonin enhances drought tolerance by regulating the coordination of C and N metabolism. The results will contribute to further understanding of the role played by melatonin in alleviating drought stress.

## Materials and Methods

### Plant Materials and Treatments

A pot experiment was conducted from May to August 2020 at the rainproof shed of the Maize Experimental Station of Northwest A&F University, Shaanxi, China. Maize (*Z. mays* L. “Shaandan 609”) seeds were sown in plastic pots of uniform size (diameter 26 cm and depth 38 cm), each filled with 15 kg air-dried soil and 10 g compound fertilizer containing 24% N, 6% P_2_O_5_, and 10% K_2_O. The soil water content is expressed as a percentage maximum of pot capacity (Ogbaga et al., 2014). All plants were watered to 85% before the seven-leaf stage. Afterward, half of the pots were exposed to drought conditions. During this period, all pots were sprayed with either melatonin (100 μM) or distilled water at 8 p.m. every day. The sprayed melatonin solution was prepared by dissolving 1.15 g melatonin powder in 25 ml ethanol as a stock solution. Subsequently, a melatonin solution of the desired concentration was obtained by further dilution with distilled water, including 0.05% (v/v) Tween-20 as a surfactant. In the present study, the maize seedlings were subjected to four treatment regimes: (1) distilled water pretreatment plus ample water (Control, CK); (2) 100 μM melatonin plus ample water (MT); (3) distilled water pretreatment plus drought (DS); (4) 100 μM melatonin plus drought (DS + MT). The melatonin concentration (100 μM) applied in this study was chosen based on a study by Guo et al. (2020a). The experiment was stopped when the soil water content decreased to 50%, i.e., after drought for 6 days. At the end of the treatments, the fully expanded third leaf from the top of the plant was gathered, rapidly frozen in liquid nitrogen, and stored at –80°C for the following measurements.

### Plant Growth Attributes

The plant leaf was measured with a tape measure on the last day of the experiment to calculate the leaf area, as described by Ahmad et al. (2019): leaf area = leaf length × maximum leaf width × 0.75. The aboveground plant parts from each group were sampled, and their fresh biomass was determined. Then, the aboveground parts of the maize plant were oven-dried at 105°C for 45 min and then maintained at 80°C for 48 h to obtain a stable dry weight. The amount of chlorophyll in the fully expanded third leaf from the top was determined using a SPAD-502 Plus chlorophyll meter (Plus, Konica Minolta, Japan).

To evaluate the water stress effects, measurements of the relative water content of leaves (RWC) were performed based on the method of Li et al. (2014) with some modifications. Briefly, a total of 1 g of fresh leaves tissue was immediately excised and weighed (fresh weight, WF), and again weighed after floating leaf segments on the water for 12 h in the dark (saturated weight, WS) and after oven-drying at 85°C for 24 h to a constant weight (WD). The RWC was calculated as follows:


(1) RWC(%)=[(WF-WD)/(WS-WD)]× 100

### Determination of Gas Exchange Parameters and Chlorophyll Fluorescence

Gas exchange parameters, such as photosynthetic rate (Pn), intercellular CO_2_ concentrations (Ci), stomata conductance (Gs), and transpiration rates (Tr) were recorded between 10:00 a.m. and 12:00 a.m. on the fully expanded third leaf from the top with an LI-6400XT portable photosynthesis system (LI-COR, Biosciences, Lincoln, NE, United States). During the measurement period, the photosynthetic photon flux density (PPFD) was controlled at 1,200 μmol m^–2^ s^–1^ (light saturation), the blocking temperature was at 25°C, the CO_2_ concentration in the air entering the leaf chamber was at 400 μmol mol^–1^, and the relative humidity was at 50–70%, according to Dai et al. (2020). Each treatment was replicated three times.

The eighth leaf was selected to evaluate chlorophyll fluorescence *via* the saturation pulse technique, using the Pulse Amplitude Modulated system (Dual-PAM-100, Heinz Walz, Effeltrich, Germany). The maximum efficiency of PSII photochemistry (Fv/Fm), quantum efficiency of PSII [Y(II)], quantum yield regulated energy dissipation of PSII [Y(NPQ)], the quantum yield of non-regulated energy dissipation of PSII [Y(NO)], and photosynthetic electron flows through PSII [ETR(II)] were imaged and calculated after adaptation in the dark for 30 min (Guo et al., 2020b).

### Quantification of Carbohydrates

The amounts of sucrose, glucose, and fructose were determined by using high-performance liquid chromatography (HPLC) (Xu et al., 2020). Briefly, a frozen leaf sample (1.0 g) was ground in 5 ml of extraction buffer (ethanol: chloroform: water = 12: 5: 3), and transferred to a centrifuge tube containing 25 ml of ultrapure water. Then, the mixture was heated to 80°C in a water bath for 1 h. After cooling to room temperature, the mixture was centrifuged at 10,000 × *g* for 15 min. The supernatant was filtered into a 50 ml volumetric flask, and the volume was brought to 50 ml with ultrapure water. This solution was used to quantitate the sugars, and the residue was used to quantitate the starch.

Starch was quantified in leaves with the Anthrone method as described by Hansen and Moller (1975). The starch was extracted with 20 ml of deionized water and heated in boiling water for 15 min using the residue obtained in the above extraction process. Then, the residue was extracted with 2 ml of 9.2 M perchloric acid and heated in boiling water for 15 min. After the mixture was centrifuged at 4,000 × *g* for 15 min, the supernatant was brought to a final volume of 50 ml with distilled water. Then, 2.0 ml of the supernatant was mixed with 10 ml of anthrone reagent (1.0 g of anthrone dissolved in 500 ml 72% sulfuric acid) and boiled for 10 min. After this treatment, the tube was rapidly cooled to room temperature, and the absorbance was measured at 630 nm.

### NO_3_^–^, NO_2_^–^, and NH_4_^+^ Measurements

The foliar NO_3_^–^, NO_2_^–^, and NH_4_^+^ were extracted from the tissue of each freeze-dried leaf by homogenizing with deionized water. The amount of NO_3_^–^ was spectrophotometrically determined at 410 nm by nitration of salicylic acid, as previously described by Cataldo et al. (1975). The amount of NO_2_^–^ was assayed by measuring the absorbance changes at 620 nm obtained by known concentrations of KNO_3_ (Barro et al., 1991). NH_4_^+^ was quantified by measuring the absorbance changes at 620 nm based on Brautigam et al. (2007), with (NH_4_)_2_SO_4_ as the standard.

### Quantification of Soluble Protein, Free Amino Acids, and Proline

First, 0.5 g of leaf tissues were ground in 5 ml pre-cooled 50 mM phosphate buffer (pH 7.8). The homogenate was centrifuged at 12,000 × *g* and 4°C for 20 min. The soluble protein concentration in the leaves was quantified using the Coomassie brilliant blue G-250 reagent according to Bradford (1976) with bovine serum albumin (BSA) as a standard. The free amino acid content was determined by the ninhydrin method (Yemm et al., 1955), with glycine as the standard. Proline was determined according to the method of Ye et al. (2015). Briefly, 0.5 g of fresh leaves were homogenized in 5 ml of 3% aqueous sulfosalicylic acid. Then, the mixtures of 2 ml of supernatant, 2 ml of ninhydrin reagent, and 2 ml of glacial acetic acid were boiled for 30 min, cooled, and centrifuged at 10,000 × *g* for 10 min. The absorbance was recorded at 520 nm, and the amount of proline was calculated according to a standard curve.

### Enzymatic Activity Assay

Nitrate reductase (NR) and nitrite reductase (NiR) were measured in maize leaves (0.5 g), which were homogenized with 2 ml buffer containing 0.1 M Tris–HCl (pH 7.5), 10 mM cysteine, 1 mM ethylene diamine tetraacetic acid (EDTA), and 5 μM flavin adenine dinucleotide (FAD). Then, the homogenate was centrifuged at 15,000 × *g* for 20 min at 4°C, and all the extraction steps were performed on ice. The activities of NR and NiR were measured based on the method of Barro et al. (1991).

To determine the activities of glutamine synthetase (GS), glutamate synthetase (GOGAT), and glutamate dehydrogenase (GDH), corn leaves (0.5 g) were grounded in 3 ml buffer containing 50 mM Tris–HCl (pH 8.0), 2 mM Mg^2+^, 2 mM DTT, and 0.4 M sucrose. Extracts were centrifuged at 10,000 × *g* for 10 min at 4°C, and all operations were performed on ice. GS activity was determined according to the description of O’Neal and Joy (1973). GOGAT was measured as described by Matoh and Takahashi (1982). The activity of GDH was assessed as per Loyola-Vargas and de Jimenez (1984).

The frozen leaf samples (0.5 g) were extracted in 5 ml 100 mM Tris–HCl buffer (pH 7.0) containing 5 mM MgCl_2_, 2 mM EDTA-Na_2_, 2 mM dithiothreitol (DTT), 2% β-mercaptoethanol, 0.2% BSA, and 2% polyvinylpolypyrrolidone (PVP), and the homogenates were centrifuged at 10,000 × *g* and 4°C for 10 min. All the steps were performed on ice. After centrifugation, the supernatant was analyzed to determine if sucrose phosphate synthase (SPS), sucrose synthase (SuSy), acid invertase (AI), and alkaline invertase (NI) were present according to the method of Hu et al. (2020).

To determine ADP glucose pyrophosphorylase (AGPase) activity, 0.5 g maize leaf tissues were mixed with 50 mM HEPES-NaOH buffer, then centrifuged at 4°C for 10 min at 10,000 × *g*. The supernatant was used to determine the activity of AGPase according to Schaffer and Petreikov (1997). All extractions were carried out on the ice.

Citrate synthase (CS) activity in the frozen leaf samples (0.5 g) was extracted with 5 ml 200 mM Tris–HCl buffer (pH 8.2) containing 0.1% Triton X-100 and 10 mM erythorbic acid (Terrier et al., 2001). The samples were grounded in an ice bath, and the homogenates were centrifuged at 5,000 × *g* for 20 min at 4°C. The supernatant was used to determine the activity of CS according to Johnson et al. (1994).

The frozen leaf samples (0.5 g) were grounded with 5 ml 100 mM phosphate buffer (pH 7.2–7.4) in an ice bath, and the homogenates were centrifuged at 3,000 × *g* and 4°C for 20 min. The supernatant was then used for the enzymatic assay. The activity of phosphoenolpyruvate carboxylase (PEPC) was determined using a detection kit (Jingkang, Shanghai).

### RNA Extraction and Real-Time Quantitative PCR Assay

The frozen leaf samples (approximately 100 mg) were grounded into powder under liquid nitrogen, and the total RNA of the different treatments was extracted using TRIzol reagent (Thermo Fisher, MA, United States). Then, 2 μg of total RNA was reverse transcribed according to the instructions of the reagent manufacturer (HiScript II Q-RT SuperMix for qPCR, Vazyme, China). The primer sequences for RT-PCR were designed by Primer-BLAST (GenBank, NCBI) and are shown in Table 1. qRT-PCR was performed using the CFX96 real-time PCR detection system (Bio-Rad, Hercules, CA, United States) with SYBR Green I (Bio-Rad). The two-step PCR method was performed, and the PCR conditions were as follows: pre-denaturation at 95°C for 30 s, 40 cycles of 95°C for 5 s, and 60°C for 30 s. The results were calculated according to the 2^–ΔΔ^ ^CT^ method (Mohd et al., 2011). Three biological replicates were performed, and β-actin was used as an internal reference gene.

**TABLE 1:** Primers used for real-time PCR (RT-PCR) amplification.

Genes	Sense primer	Anti-sense primer
*ZmSh1*_sucrose synthase	GATGCCCTGTTTGATAGTGA	ATCGTCGTGCCCTTGTAG
*Zmsps1*_sucrose phosphate synthetase	CCAGCGGCATGTGAATTTGAT	CACCAGTATAGTTAGCAGTGTCC
*ZmAgp1*_ADP-glucose pyrophosphorylase	GTTGTTTGAGGAGCATAAT	ACAGATAAGCCTGAACCC
*Zmcts1*_citrate synthase	TGCTCACAGTGGAGTTTTGC	AACACTCTTCGGCCTCTCAA
*ZmPEPC*_phosphoenolpyruvate carboxylase	GAAGACACGCTCATCCTCACC	CAGTTCGGCATTTCCATCC
*ZmRCA1*_RuBisCo activase	GCAAAGGCCAGGGAAAATCG	ATGTTCATCAGGGTGGCGTT
*ZmrbcS*_RuBisco small subunit	GCAGGAGGCCATCAAATCCT	AAGCAAGCAAAGGGTACGGT
*ZmrbcL*_RuBisCo large subunit	TGATGGGACAACCACTTCGG	GTACAGCCACCACCTACGAT
*ZmGln1-3*_cytosolic glutamine synthetase	CGAAGCGATTGCAAAGCCATTG	GTTCTGTTTTGGCACACCAC
*ZmGS2*_plastidic glutamine synthetase	TGTGAAGCAGCTGAAGGATG	CGTATCCGAATATCCGATGAA
*Zmgdh1*_glutamate dehydrogenase	GTCATAAACAAGGATAATGCTAACG	CCAGTATGTCGGGGAGGAT
*Zmfgs1*_glutamate synthase	CTGATCGTTCTGAAGCACCT	AGCAGACATACGGAGACCAT
*ZmNR*_nitrate reductase	ATGATCCAGTTCGCCATCTC	GTCCGTGGTACGTCGTAGGT
*ZmNiR*_nitrite reductase	CTTCATGGGCTGCCTCAC	CGCTTGACGAAGGTCCTACT
*ZmActin*	CCATCACTGCCACACAGAAAAC	AGGAACACGGAAGGACATACCAG

### Statistical Analysis

ANOVA was performed for the results using SPSS 25.0 software, and then Duncan’s multiple range test was carried out, with *P* < 0.05 indicating a significant difference. A SigmaPlot 10.0 was used to draw the figures. All the values are presented as the mean ± SD.

## Results

### Effects of Melatonin on Maize Growth Under Drought Stress

**FIGURE 1:**
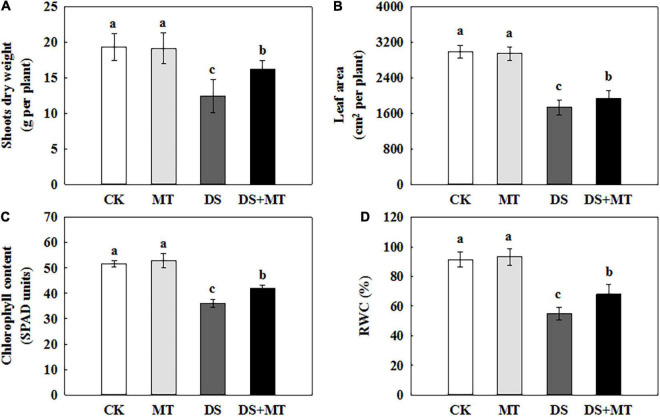
Effects of drought and exogenous melatonin on plant growth, chlorophyll content, and relative water content (RWC) in leaves of maize. **(A)** Shoot dry weight, **(B)** whole plant leaf area, **(C)** the chlorophyll content in leaves, and **(D)** the leaf relative water content. Values are the averages of three replicates ± SD. Different letters indicate significant differences according to Duncan’s multiple range tests (*P* < 0.05).

In the present study, we evaluated the effects of MT, drought stress, and their combination on the growth of maize to understand the role of melatonin in drought tolerance in maize plants. As shown in Figure 1, there were no significant effects on maize seedling growth between melatonin-treated and non-treated under the well-irrigated conditions. The water deficit caused a significant inhibition of plant growth, with the aboveground biomass accumulation and leaf area of non-treated maize seedlings decreasing by 36.0 and 42.0%, respectively, compared with control (Figures 1A,B). In comparison, exogenous melatonin application mitigated the drought stress, and the aboveground biomass accumulation and leaf area of melatonin-treated seedlings increased by 30.6 and 11.5%, respectively, compared with that of the non-treated seedlings (Figures 1A,B). Drought stress resulted in a sharp decrease in chlorophyll and the RWC, while the application of exogenous melatonin reversed these trends to some extent (Figures 1C,D). In relation to control, water deficit substantially reduced chlorophyll and the RWC by 30.17 and 40.19%, respectively. However, melatonin treatment caused significant recovery of chlorophyll and the RWC by 16.93 and 24.41%, respectively, compared with the drought stress treatment.

### Effects of Melatonin on Stomatal Behavior Under Drought Stress

**FIGURE 2:**
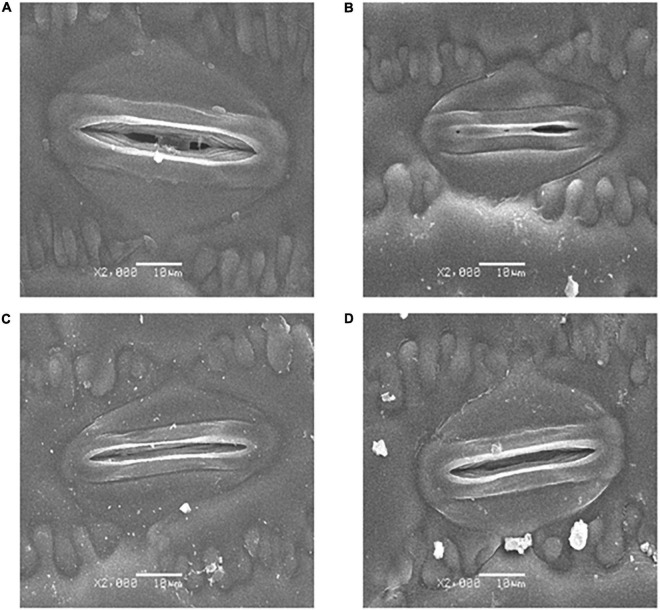
Effects of drought and exogenous melatonin on stomata in leaves. **(A)** Stomata from well-watered plants. **(B)** Stomata from drought-treated plants. **(C)** Stomata from well-watered plants that were also treated with 100 μM melatonin. **(D)** Stomata from drought-treated plants that were also treated with 100 μM melatonin. Magnification 2000 X, scale bars = 10.0 μm.

**FIGURE 3:**
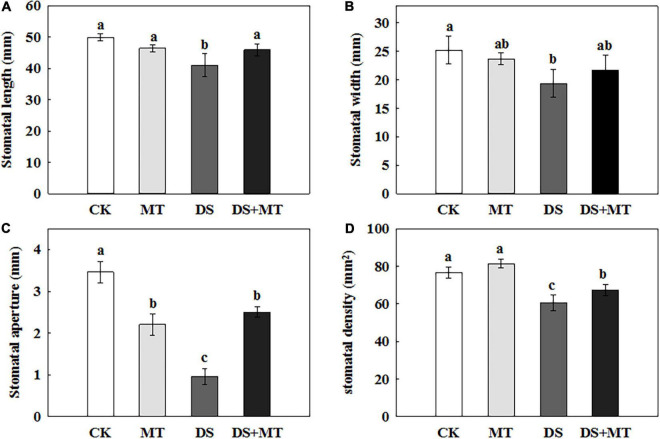
Effects of drought and exogenous melatonin on stomatal characteristics in leaves of maize. **(A)** Stomatal length, **(B)** stomatal width, **(C)** stomatal aperture, and **(D)** stomatal density. Values are the averages of three replicates ± SD. Different letters indicate significant differences according to Duncan’s multiple range tests (*P* < 0.05).

The SEM stomatal images showed that the stomata were almost completely closed by drought stress (Figure 2B), stomatal aperture exhibited a 72.3% decrease in comparison with control (Figure 3C). Moreover, drought stress also led to the stomata being shorter, narrower, and thinner. The stomatal length, width, and density in the plants that underwent drought stress alone were 82.3, 76.7, and 79.1% of that of control, respectively (Figures 3A,B,D). Compared with the plants that received limited water, the stomata remained partially open in the melatonin-treated maize under drought stress (Figure 2D). Correspondingly, the stomatal aperture of melatonin-treated plants was 1.3-fold higher than that of stressed plants (Figure 3C). The melatonin-treated plants had longer and wider stomata under drought stress. The stomatal length and width in maize seedlings treated with melatonin were increased by 11.9 and 12.0%, respectively, in contrast to the drought stress-treated plants (Figures 3A,B). In addition, the stomatal length and width were not affected by the application of melatonin compared with the well-watered plants (Figures 3A,B), but the stomatal density of melatonin-treated plants under soil drought stress was less than that of control (Figure 3D). Under well-watered conditions, the application of melatonin decreased the stomatal aperture by 36.2%, but it had no effect on other characteristics of stomata (Figure 3).

### Effects of Melatonin on Photosynthesis of Plants Under Drought Stress

**FIGURE 4:**
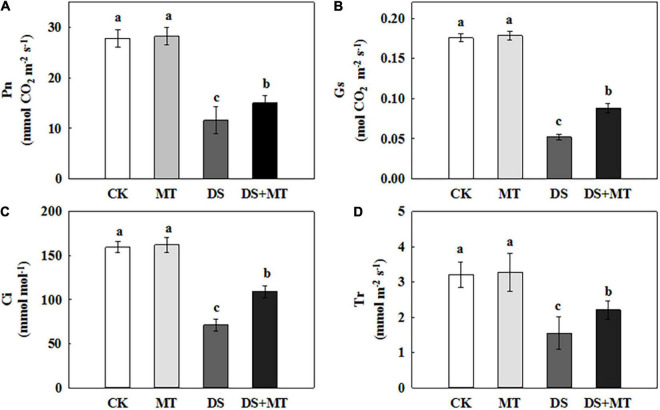
Effects of drought and exogenous melatonin on gas exchange parameters in leaves of maize. **(A)** Net photosynthetic rate (Pn), **(B)** stomatal conductance (Gs), **(C)** intercellular CO_2_ concentration (Ci), and **(D)** transpiration rate (Tr). Values are the averages of three replicates ± SD. Different letters indicate significant differences according to Duncan’s multiple range tests (*P* < 0.05).

**FIGURE 5:**
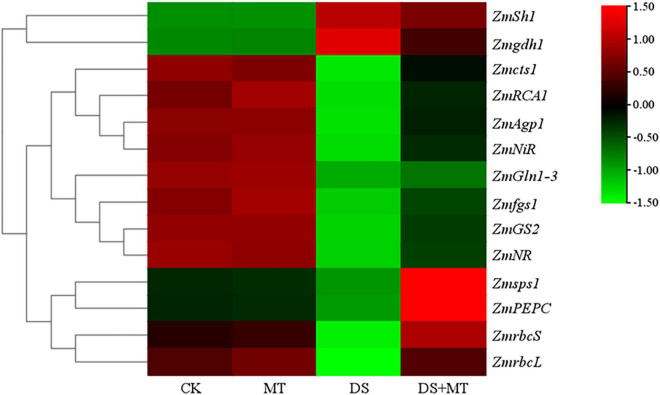
A hierarchical clustering heat map of genes encoding enzymes involved in the processes of photosynthetic carbon assimilation, carbon metabolism, and nitrogen assimilation. Normalization of expression values was performed by log10 (FPKM). For each gene, low expression is indicated by green; high expression is indicated by red.

Under well-watered conditions, the application of exogenous melatonin resulted in no obvious change in the ability to photosynthesize (Figure 4). After 7 days of drought stress, the Pn, Ci, Gs, and Tr were decreased by 58.3, 55.3, 70.4, and 51.7%, respectively, compared with control (Figure 4). In contrast, the exogenous melatonin-treated plants exhibited fewer negative effects of drought stress, with a decrease of only 46.0, 31.5, 50.0, and 31.2% for Pn, Ci, Gs, and Tr, respectively, compared with control (Figure 4). These data suggest that there was an increased photosynthetic performance for the melatonin-treated plants compared with the non-treated plants under drought stress. In addition, the melatonin treatment increased the Rubisco activity from 25.5 to 38.31 mg g^–1^ h^–1^ FW, and the change in Rubisco activity was parallel with the expression of *ZmRCA1*, *ZmrbcL*, and *ZmrbcS* (Table 2 and Figure 5). These results further support the ability of melatonin-treated plants to maintain photosynthetic C assimilation during drought stress.

**TABLE 2:** Effects of drought and exogenous melatonin on PSII chlorophyll fluorescence parameters and Rubisco activity in leaves of maize.

Parameters	CK	MT	DS	DS + MT
Fv/Fm	0.815 ± 0.04^a^	0.817 ± 0.05^a^	0.758 ± 0.02^c^	0.797 ± 0.03^b^
Y(II)	0.431 ± 0.04^a^	0.438 ± 0.02^a^	0.226 ± 0.03^c^	0.341 ± 0.02^b^
Y(NPQ)	0.296 ± 0.01^c^	0.286 ± 0.02^c^	0.453 ± 0.03^a^	0.369 ± 0.02^b^
Y(NO)	0.273 ± 0.02^b^	0.276 ± 0.01^b^	0.320 ± 0.02^a^	0.290 ± 0.04^b^
ETR(II)	38.7 ± 1.46^a^	38.6 ± 1.56^a^	20.3 ± 1.67^c^	31.2 ± 1.55^b^
Rubisco activity (mg g^–1^ h^–1^ FW)	30.63 ± 1.88^b^	31.82 ± 1.45^b^	25.52 ± 1.48^c^	38.31 ± 1.01^a^

*The values are the averages of three replicates ± SD. Different letters indicate significant differences according to Duncan’s multiple range tests (P < 0.05).*

*Fv/Fm, quantitative values of maximum PSII yield; Y(II), effective quantum yield of PSII; Y(NPQ), quantum yield of regulatory energy dissipation; Y(NO), quantum yield of non-regulatory energy dissipation; ETR(II), electron transport rate of PSII.*

To further investigate the alterations of photosynthesis in maize plants exposed to soil drought stress, multiple chlorophyll fluorescence parameters, such as Fv/Fm, Y(II), Y(NPQ), Y(NO), and ETR(II) were calculated (Table 2). The results of fluorescence measurement showed that the application of melatonin did not change the chlorophyll fluorescence parameters under the well-irrigated condition (Table 2). Compared with control, drought stress markedly reduced the Fv/Fm, Y(II), and ETR(II) by 7.0, 47.6, and 47.5%, respectively. However, melatonin application resulted in the significant reversal of the Fv/Fm, Y(II), and ETR(II) by 5.1, 50.9, and 53.7%, respectively. Moreover, the opposite effects were observed in Y(NPQ) and Y(NO), and the Y(NPQ) and Y(NO) of non-irrigated plants not treated with melatonin were 53.0 and 17.2% higher than the CK seedlings. Compared with the untreated seedlings that underwent water restriction, melatonin treatment significantly decreased the Y(NPQ) and Y(NO) levels, with Y(NO) being decreased to the normal level.

### Effects of Melatonin on Carbon Metabolites Under Drought Stress

**FIGURE 6:**
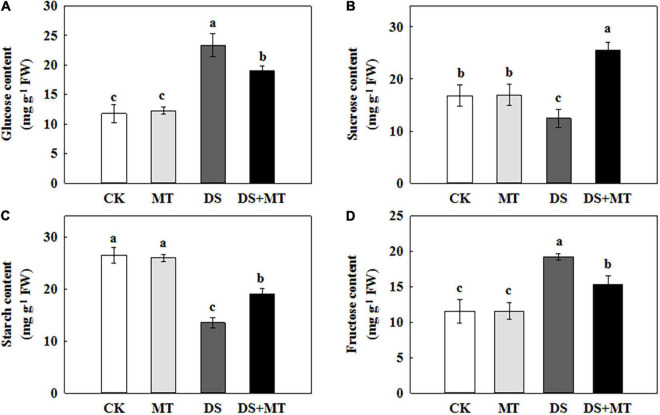
Effects of drought and exogenous melatonin on the sugar content in leaves of maize. **(A)** Glucose content, **(B)** sucrose content, **(C)** starch content, and **(D)** fructose content. Values are the averages of three replicates ± SD. Different letters indicate significant differences according to Duncan’s multiple range tests (*P* < 0.05).

Carbon metabolism is tightly linked with photosynthesis. To elucidate how melatonin regulates C metabolic homeostasis in maize under drought stress, we further measured the amount of carbohydrate and analyzed the activities of C-metabolizing enzymes in different treatments (Table 3 and Figure 6). Exogenously applied melatonin did not affect the amounts of soluble sugar, sucrose, starch, or fructose under normal conditions (Figure 6). In relation to control, there was a remarkable decrease in sucrose and starch in maize leaves (74.2 and 51.1% of control plants), when subjected to drought (Figures 6B,C). In contrast, melatonin treatment substantially increased the concentrations of sucrose and starch by 105.9 and 40.8%, respectively, compared with the drought-stressed plants (Figures 6B,C). Drought stress increased glucose and fructose levels by 97.9 and 66.4%, respectively, compared with control (Figures 6A,D). However, after 7 days of drought stress, the glucose and fructose in maize seedlings treated with melatonin were reduced by 18.7 and 20.5%, respectively, in contrast to the non-treated plants (Figures 6A,D).

**TABLE 3:** The effects of drought and exogenous melatonin on C-related enzymatic activities in leaves of maize.

Parameters	CK	MT	DS	DS + MT
SS activity (mg g^–1^ h^–1^FW)	17.31 ± 1.02^a^	16.96 ± 2.09^a^	28.92 ± 2.10^c^	22.29 ± 1.99^b^
SPS activity (mg g^–1^ h^–1^FW)	63.19 ± 4.23^b^	64.17 ± 3.11^b^	46.84 ± 3.11^c^	75.19 ± 1.78^a^
INV activity (mg g^–1^ h^–1^FW)	5.48 ± 0.30^a^	5.82 ± 0.86^a^	15.00 ± 1.62^c^	9.43 ± 1.26^b^
AGPase activity (mg g^–1^ h^–1^FW)	24.04 ± 1.40^a^	25.09 ± 1.02^a^	16.56 ± 0.93^c^	20.71 ± 2.34^b^
PEPC activity (μmol CO_2_ mg^–1^ h^–1^)	53.27 ± 4.47^b^	55.63 ± 3.40^b^	41.56 ± 5.08^c^	65.77 ± 6.15^a^
CS activity (mg g^–1^ h^–1^FW)	19.14 ± 1.63^c^	19.87 ± 1.27^c^	11.39 ± 1.26^b^	15.22 ± 1.28^a^

*The values are the averages of three replicates ± SD. Different letters indicate significant differences according to Duncan’s multiple range tests (P < 0.05).*

*SS, sucrose synthase; SPS, sucrose phosphate synthetase; INV, invertase; AGPase, ADP-glucose pyrophosphorylase; PEPC, phosphoenolpyruvate carboxylase; CS, citrate synthase.*

Under normal growth conditions, all the C-metabolizing enzymatic activities that were evaluated in maize leaves were not altered by exogenous melatonin (Table 3). Compared with the control, soil water deficit caused considerable increases in SS and INV activity in melatonin-treated (1.3-fold and 1.7-fold of the control plants) and -untreated (1.7-fold and 2.7-fold of the control plants) maize seedlings, but the extent of increase of melatonin treatment was significantly lower than that of drought stress treatment (Table 3). In contrast to the control plants, water restriction resulted in a drastic decline of the activities of SPS, AGPase, PEPC, and CS by 34.9, 31.1, 22.0, and 40.5%, respectively. Compared with the drought stress treatment, the activities of SPS, AGPase, PEPC, and CS in melatonin-treated plants were increased by 60.5, 25.1, 53.6, and 33.6, respectively, with the activities of SPS and PEPC being notably higher than those of control. Furthermore, we found that the melatonin-mediated modulation of C-metabolizing enzymes was due to the induced expression of key genes encoding these enzymes, namely, *ZmSh1*, *ZmAgp1*, *Zmcts1*, *Zmsps1*, *and ZmPEPC* (Figure 5).

### Effects of Melatonin on Nitrogen Metabolism Under Drought Stress

**FIGURE 7:**
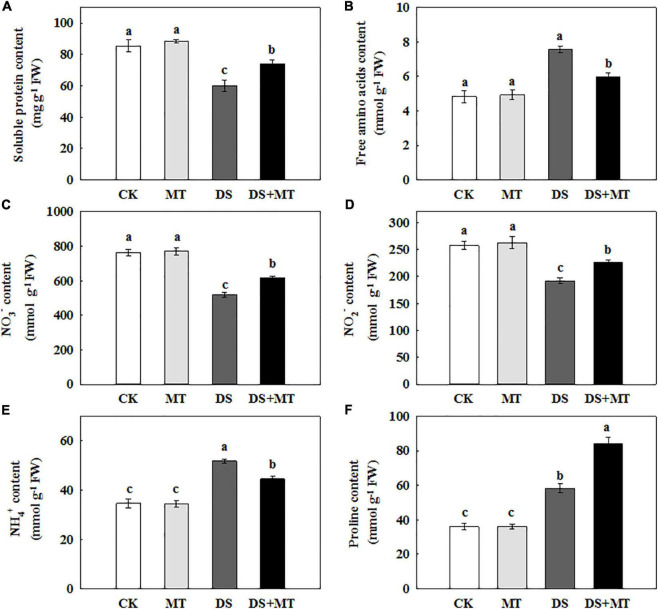
Effects of drought and exogenous melatonin on soluble protein, free amino acid, inorganic N compounds, and proline content in the leaves of maize. **(A)** Soluble protein content, **(B)** free amino acid content, **(C)** nitrate $\left({\text{NO}}_{3}^{\text{-}}\right)$ content, **(D)** nitrite $\left({\text{NO}}_{2}^{\text{-}}\right)$ content, **(E)** ammonium $\left({\text{NO}}_{4}^{\text{-}}\right)$ content, and **(F)** proline content. Values are the averages of three replicates ± SD. Different letters indicate significant differences according to Duncan’s multiple range tests (*P* < 0.05).

Nitrogen metabolism is closely associated with chlorophyll fluorescence and C assimilation. Under the well-irrigated condition, exogenous melatonin application resulted in no remarkable changes in the amounts of primary N metabolites (Figure 7). Water stress led to a significant reduction of soluble protein, NO_3_^–^, and NO_2_^–^ by 29.8, 31.9, and 25.5%, respectively, compared with control. However, in drought-stressed plants, the melatonin treatment increased soluble protein, NO_3_^–^, and NO_2_^–^ by 23.4, 18.9, and 15.1%, respectively (Figure 7). Under water deficit, free amino acids, NH_4_^+^, and proline in untreated plants were increased by 56.1, 49.5, and 61.4%, while in melatonin-treated plants, these were increased by 23.2, 28.3, and 132.9%, respectively (Figure 7).

The activities of the six N metabolic enzymes were hardly affected by exogenous melatonin under the well-watered condition. Due to the suppression of the expression of *ZmNR*, *ZmNiR*, *ZmGln1-3* and *ZmGS2*, and *Zmfgs1*, which are the key genes encoding NR, NiR, GS, and GOGAT in drought-stressed plants, these enzymatic activities decreased by 36.8, 37.3, 40.4, and 31.9%, respectively, compared with that in the control plants (Table 4 and Figure 5). Exogenous melatonin-treated leaves exhibited higher NR, NiR, GS, and GOGAT activities than the drought-stressed leaves, and the expression of the respective genes was also higher in the melatonin-treated leaves compared with the drought-stressed plants (Table 4 and Figure 5). In contrast, withholding irrigation significantly increased the foliar GDH amination and GDH deamination activities compared with the control plants, with an average increase of 2.2- and 1.5-time (Table 4). The qRT-PCR analyses indicated that the transcript levels of *Zmgdh1*, a key gene encoding GDH, were also dramatically induced by drought stress (Figure 5). However, the GDH amination and deamination activities (79.4 and 68.1% of drought stressed plants, respectively) and *Zmgdh1* expression were notably inhibited by the addition of 100 μM melatonin compared with the drought stress treatment (Table 4 and Figure 5).

**TABLE 4:** The effects of drought and exogenous melatonin on N-related enzymatic activities in leaves of maize.

Parameters	CK	MT	DS	DS + MT
NR activity (mmol NO_2_^–^ mg^–1^ h^–1^ FW)	0.37 ± 0.01^a^	0.34 ± 0.02^a^	0.23 ± 0.01^c^	0.27 ± 0.01^b^
NiR activity (mmol NO_2_^–^ mg^–1^ min^–1^ FW)	0.51 ± 0.03^a^	0.53 ± 0.01^a^	0.32 ± 0.01^c^	0.43 ± 0.02^b^
GS activity (mg g^–1^ h^–1^ FW)	70.46 ± 5.81^a^	71.57 ± 5.86^a^	42.11 ± 4.62^c^	56.62 ± 4.26^b^
GOGAT activity (mmol mg^–1^ Prot min^–1^)	3.98 ± 0.18^a^	4.08 ± 0.15^a^	2.71 ± 0.13^c^	3.40 ± 0.15^b^
NAD-GDH activity (nmol mg^–1^ Prot min^–1^)	4.20 ± 0.24^c^	4.25 ± 0.31^c^	9.38 ± 0.13^a^	6.39 ± 0.21^b^
NADH-GDH activity (nmol mg^–1^ Prot min^–1^)	11.09 ± 0.45^c^	11.13 ± 0.37^c^	16.52 ± 0.52^a^	13.11 ± 0.33^b^

*The values are the averages of three replicates ± SD. Different letters indicate significant differences according to Duncan’s multiple range tests (P < 0.05).*

*NR, nitrate reductase; NiR, nitrite reductase; GS, glutamine synthetase; GOGAT, glutamate synthetase; NAD-GDH, deaminating glutamate dehydrogenase; NADH-GDH, aminating glutamate dehydrogenase.*

## Discussion

### Exogenous Melatonin Enhanced Photosynthetic Carbon Assimilation by Promoting Stomatal Opening Under Drought Stress

Water deficit stress severely inhibits plant growth and development by affecting various aspects of plants physiology and biochemistry (Meng et al., 2014; Guo et al., 2020a; Gupta et al., 2020). Various types of research have demonstrated that exogenously applied melatonin can enhance drought tolerance in plants (Guo et al., 2020a; Hu et al., 2020; Khattak et al., 2021). Our results showed that the growth of maize was critically suppressed by water deficit because the drought-stressed plants exhibited lower values of leaf area and shoot dry weight compared with the control plants (Figure 1). In contrast, foliar-applied melatonin mitigates plant growth inhibition caused by drought stress, indicating that the exogenous melatonin application increased the tolerance to water deficit in plants (Figure 1). Moreover, we also observed that there was a reduction of the chlorophyll content and RWC after 7 days of drought stress, while melatonin treatment attenuated the decrease in chlorophyll content and RWC (Figure 1). Similar research results were observed in previous reports (Huang et al., 2019; Dai et al., 2020). These consequences may be due to the application of melatonin, which can facilitate photosynthesis.

Our results indicate that the water deficit significantly decreased photosynthetic activity in maize (Figure 4). Photosynthesis is the principal process of capturing light energy to synthesize carbohydrates, and it is closely related to the growth of plants. However, photosynthesis is sensitive to drought stress, and a water deficit notably inhibits photosynthesis in many plants (Velikova et al., 2018; Zhou et al., 2019; Sharma et al., 2020). In general, the decrease in photosynthetic activity is limited by the reduction in CO_2_ diffusion to the chloroplast, which is induced by stomatal closure (Liu et al., 2013; Ye et al., 2016). The closure of stomata restricts the mesophyll transport of CO_2_, resulting in a decrease in the concentration of CO_2_ in the intercellular airspaces of leaves. Low intercellular carbon dioxide (Ci) will decrease the activities of key enzymes, such as Rubisco to limit the photosynthesis rate (Flexas et al., 2006; Chaves et al., 2009; Zhong et al., 2019). As expected, our results confirmed that drought caused the stomata to close almost completely (Figure 2). The Ci and Pn level, and the activity of Rubisco, and the expression of several genes encoding key enzymes in Rubisco also decreased under the drought conditions (Figures 4, 5 and Table 2). These results further support the conclusion that the stomatal closure in water-stressed plants may be one of the reasons for the decrease in photosynthesis. However, the melatonin treatment increased the stomatal aperture and partially opened stomata under a water deficit (Figure 2). In addition, melatonin significantly increased stomatal density and stomatal length compared with water deficit stress (Figure 3). We speculate that in response to drought stress, an optimization strategy for stomatal structure and distribution would be beneficial. Similar research results were obtained in rape, with low stomatal width and high stomatal density observed in rape plants that experienced drought (Dai et al., 2020). Correspondingly, the higher Rubisco activity and Pn value were observed in melatonin-treated plants compared with the drought-stressed plants (Figures 3, 4 and Table 2), indicating that melatonin increased the C fixation and photosynthetic activity in maize plants under drought stress.

Chlorophyll fluorescence is an important indicator that can be used to characterize the photosynthetic capacity and energy conversion efficiency of PSII in plants (Mathur et al., 2019). Many studies have demonstrated that severe or long-term water deficit leads to photo-inhibition in the PSII reaction center (Huang et al., 2019; Zhou et al., 2019). Consistent with these findings, a large decrease in Fv/Fm, Y(II), and ETR(II) was observed in drought-stressed plants (Table 2). Fv/Fm, Y(II), and ETR(II) decreased, while Y(NPQ) and Y(NO) increased, indicating that drought stress-induced severe damage to the PSII complexes in maize seedlings. This was attributed to the fact that the limitation of ambient CO_2_ diffusion to the site of carboxylation resulted in a relative excess of light energy and electron sinks, and led to photo-inhibition or photo-oxidation (Atkin and Macherel, 2009; Zhong et al., 2018). However, exogenous melatonin treatment can increase the photosynthetic efficiency and protect the maize plants from photo-inhibition caused by drought, because among plants exposed to drought stress, those treated with exogenous melatonin exhibited enhanced Fv/Fm, Y(II), and ETR(II), and decreased Y(NPQ) and Y(NO) levels (Table 2). Consistent with the current results, a previous study demonstrated that melatonin-treated tomato plants displayed significantly increased Fv/Fm and ΦPSII compared with the non-treated plants under water deficit conditions (Liu et al., 2015). Additionally, the application of melatonin produces a protective effect on chlorophyll (Campos et al., 2019; Li Z. et al., 2021). The amount of chlorophyll in melatonin-treated maize plants was higher as compared with the non-treated plants under drought stress in this study (Figure 1C), confirming that exogenous melatonin slows damage to the photosynthetic apparatus.

### Exogenous Melatonin Mitigated Drought Stress by Maintaining Carbohydrate Balance

In view of the inhibition of the photosynthetic capacity by soil water restriction, we observed that there was low carbohydrate synthesis in the drought-stressed maize plants (Figure 6). Plant growth and carbohydrate metabolism are closely linked because carbohydrates are the structural components and the energy source for the production and maintenance of biomass (Song et al., 2020). In higher plants, carbohydrates, such as sucrose and starch are created in photosynthetically active leaves (sources) and then exported to support sinks, which allow leaf expansion, and stem and root growth (Adams et al., 2013; Song et al., 2020). In the present study, we found that drought stress-induced a pronounced decrease in the activity and gene transcription of the main enzymes (AGPase and SPS) involved in starch and sucrose synthesis, leading to a lower starch and sucrose level in the leaves (Figures 5, 7 and Table 3). This phenomenon is attributed to the growth inhibition observed in drought-stressed plants. Similar results were reported in soybean (Du et al., 2020). Along with the enhancement of the activity and gene transcription of AGPase and SPS (Table 3 and Figure 5), foliar spraying of melatonin facilitated starch and sucrose biosynthesis in maize leaves compared with the drought-stressed plants (Figure 6). These results demonstrated that melatonin treatment supports the growth of maize plants by the accumulation of additional photosynthates. The positive correlation between melatonin and carbohydrate synthesis was confirmed in the previous studies (Campos et al., 2019; Hu et al., 2020).

In addition, we observed that the levels of glucose and fructose were significantly enhanced in the water deficit-stressed maize plants compared with the control plants (Figure 6). Higher concentrations of glucose and fructose in the leaves of drought-stressed plants might be attributed to the enhancement of SS and INV activities (Table 3) because both enzymes can fragment sucrose into hexose sugars (Gandin et al., 2009). This phenomenon also partially explains why the sucrose content in the leaves decreased under a water deficit. Another possible reason for the increase in glucose and fructose level is that drought stress inhibits the tricarboxylic acid (TCA) cycle. It has been reported that water restriction repressed the activity of the TCA cycle, which would reduce the oxidation of glucose and result in a depletion of the ATP pool (Nguyen et al., 2010; Hu et al., 2020). Our results are in agreement with this interpretation, as we found that drought stress decreased the activity and gene transcription of PEPC and CS, and increased the amounts of glucose and fructose in the leaves, compared with the control plants. However, recent research indicated that melatonin was involved in regulating the TCA cycle and could enhance energy production in water-stressed anthers (Hu et al., 2020). Our work further confirmed the protective role of melatonin on energy production in maize under drought stress. Exogenously applied melatonin increased energy production in drought-stressed plants (Figure 6). This increased energy can be further used for plant growth, thus promoting the growth of plants subjected to drought stress. Furthermore, the enhancements in the activities of the TCA cycle induced by melatonin under drought stress will provide more C skeletons and energy for the biosynthesis of downstream amino acids.

### Exogenous Melatonin Improved Nitrogen Metabolism Under Drought Stress

Soil water deficit often causes a decrease in the activities of N assimilation enzymes and the synthesis of N-containing compounds to disrupt N metabolism (Zhong et al., 2018; Xie et al., 2019). In this regard, several previous studies have shown that drought stress can inhibit the uptake of NO_3_^–^, resulting in a decrease in NR activity (Miranda-Apodaca et al., 2020; Ren et al., 2020). In this study, consistent with the decrease in NO_3_^–^ and NO_2_^–^ (Figure 7), drought stress triggered a marked diminution in NR and NiR activities, which reflects the decrease in the N assimilation capacity under a water deficit. Additionally, although the NR and NiR activities decreased under drought, it was also observed that NH_4_^+^ accumulated in maize leaves (Table 4 and Figure 7). This increment can be explained by the glycine oxidation in activated photorespiration (Zhong et al., 2018). Under drought stress, the expression levels of the *ZmNR and ZmNiR* genes, which encode the NR and NiR enzymes, were enhanced in the melatonin-treated plants (Table 4 and Figure 5). Correspondingly, the melatonin-treated plants under drought stress exhibited higher NR and NiR transcription and activity than untreated plants (Table 4 and Figure 5). In drought-stressed plants that were treated with melatonin, the increased NO_3_^–^ and NO_2_^–^ levels were in accordance with the increase in NR and NiR activities (Figure 7). In this context, induced NO_3_^–^ reduction by melatonin treatment resulted in the maintenance of the osmotic pressure in photosynthetic cells (Zhong et al., 2019). Moreover, NO_3_^–^ reduction is a process with a high energy requirement, and increased NO_3_^–^ reduction in leaves would be facilitated due to the excessive energy derived from the photosynthetic apparatus (Sunil et al., 2013; Zhong et al., 2018).

The excessive accumulation of NH_4_^+^ in plant leaves due to drought stress has a toxic effect on plants because a high level of NH_4_^+^ triggers protein extrusion and cytosolic pH disturbances (Xie et al., 2019). In plants, NH_4_^+^ must be assimilated *via* the GS/GOGAT cycle and GDH pathway into glutamine and glutamate (Liu et al., 2019). However, GDH has a lower affinity for NH_4_^+^, and the GDH pathway is markedly activated only when the GS/GOGAT cycle is restrained (Xie et al., 2019). In this study, consistent with the transcription data of genes encoding GS and GOGAT (Figure 5), there were dramatically decreased GS and GOGAT activities in the plants exposed to drought (Table 4), and this could be another important reason for the accumulation of NH_4_^+^. Those results are consistent with those reported previously (Jing et al., 2021). In contrast, our results show that melatonin mitigates the toxic effect of NH_4_^+^, because the NH_4_^+^ assimilation was notably strengthened in melatonin-treated drought-stressed plants by enhancing the activity and gene transcription of GS and GOGAT, and decreasing the GDH activity and *Zmgdh1* (Table 4 and Figure 5). This phenomenon can be explained by the enhancement of photosynthesis and the TCA cycle, which promotes the synthesis of the C skeleton and reduces the power and provides sufficient substrates and energy for the biosynthesis of amino acids. Thus, the GS/GOGAT cycle was enhanced in plants under drought, which subsequently promoted the synthesis of glutamate and other amino acids (Liang et al., 2018; Xie et al., 2019). These results indicate the positive impact of melatonin upon coordinated C assimilation and N metabolism in plants.

It was also observed that drought substantially increased the free amino acid and proline levels in the maize leaves compared with the control treatment (Figures 7B,F), and this may have occurred because N metabolism is involved in osmotic adjustment. The synthesis and accumulation of amino acids are often a strategy to enable plants to withstand adverse environmental conditions because amino acids can serve as osmotica to maintain the stability of the cellular structure and cell osmotic pressure under drought conditions (Meng et al., 2014; Zhong et al., 2018). The treatment of melatonin dramatically enhanced the levels of soluble protein and proline, and vastly decreased the free amino acid content in maize leaves under water-limiting conditions (Figure 7). These results indicate exogenously applied melatonin in drought-stressed plants regulated cell turgor by producing additional substances that regulated osmolarity, maintained membrane integrity. Stability in protein synthesis can increase the resistance of plants to stress (Georgiadou et al., 2018). Most soluble proteins are enzymes that are involved in various metabolic pathways in plants (Sun et al., 2020). Thus, they are an important index for measuring the protein damage in the process of plant metabolism and are usually positively correlated with soil drought stress tolerance (Xie et al., 2019). In our experiments, soil drought stress significantly diminished the soluble protein content, indicating that water deficit led to protein degradation and protein damage (Figure 7A). Evidence has shown that the degradation of intracellular proteins (e.g., chloroplast proteins) is an important mechanism of the N remobilization under drought stress (Ren et al., 2020). During drought stress, the abundance and activity of enzymes that control N and C metabolism are affected by the degradation of chloroplast proteins (Ishida et al., 2008). For example, drought stress-induced chloroplast proteins inactivation and degradation, resulting in an enhancement of the free amino acid content and the loss of function of plastid enzymes (e.g., GS) (Reguera et al., 2013). In our experiment, we found that the melatonin-treated plants had less proteins degradation than the non-treated plants under drought stress, along with an upregulated expression of *ZmGS2*, stabled chloroplast function, and increased capacity for N assimilation. Taken together, exogenously applied melatonin plays a positive role in the coordination of C and N metabolism under drought stress.

## Conclusion

**FIGURE 8:**
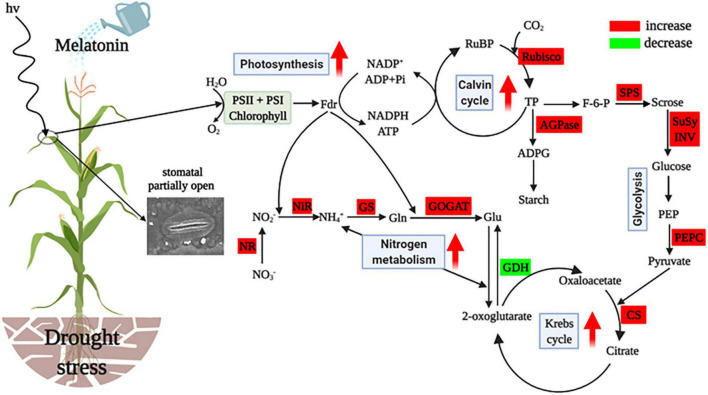
Simplified representation of exogenous melatonin affecting C and N metabolism in leaves of drought-stressed maize. Metabolism and parameters that increased or decreased it are indicated by the red or green boxes in melatonin-treated plants when compared with drought-stressed plants. The red arrows in the figure denote increased or enhanced processes. RuBP, ribulose-l,5-disphosphate; Rubisco, ribulose bisphosphate carboxylase oxygenase; TP, triose phosphate; AGPase, ADP-glucose pyrophosphorylase; ADPG, adenosine diphosphate glucose; F-6-P, fructose-6-phosphate; SPS, sucrose phosphate synthetase; SuSy, sucrose synthase; INV, invertase; PEP, phosphoenolpyravate; PEPC, phosphoenolpyravate carboxylase; CS, citrate synthase; GDH, glutamate dehydrogenase; Glu, glutamate; GOGAT, glutamate synthetase; Gln, glutamine; GS, glutamine synthetase; ${\text{NH}}_{4}^{+}$, ammonia; NiR, nitrite reductase; ${\text{NO}}_{2}^{+}$, nitrite; NR, nitrate reductase; ${\text{NO}}_{3}^{+}$, nitrate. This figure was created using BioRender (https://biorender.com/).

Based on the analysis mentioned above, the present study suggests that water deficit critically disturbs the processes of C and N metabolism, resulting in inhibited crop growth. However, exogenously applied melatonin mitigated drought stress through coordinated regulation of C and N metabolism in maize. The protective effects of exogenous melatonin on maize were mainly due to ameliorated stomatal opening and photosynthetic activity of maize, which indirectly promoted the synthesis of photosynthetic end products and energy production, and enhanced N assimilation and NH_4_^+^ detoxification, and thus consequently increased maize growth under the water restriction conditions (Figure 8). Overall, the results of this study provide valuable information for maize drought tolerance induced by melatonin and a new theoretical basis for the application of melatonin on crops grown in arid areas. Future research should explore the molecular mechanisms of functions of melatonin and the practical use of melatonin in crop production.

## Data Availability Statement

The datasets presented in this study can be found in online repositories. The names of the repository/repositories and accession number(s) can be found in the article/supplementary material.

## Author Contributions

RZ and CZ conceived and designed the experiments. CZ, HG, JW, and YW conducted the experiment and collected data for preliminary analysis. CZ, RZ, and HG further analyzed the data and wrote the manuscript. All authors reviewed and commented on the manuscript and approved the submitted version.

## Conflict of Interest

The authors declare that the research was conducted in the absence of any commercial or financial relationships that could be construed as a potential conflict of interest.

## Publisher’s Note

All claims expressed in this article are solely those of the authors and do not necessarily represent those of their affiliated organizations, or those of the publisher, the editors and the reviewers. Any product that may be evaluated in this article, or claim that may be made by its manufacturer, is not guaranteed or endorsed by the publisher.
